# Self-viewing is associated with negative affect rather than reward in highly narcissistic men: an fMRI study

**DOI:** 10.1038/s41598-017-03935-y

**Published:** 2017-07-19

**Authors:** Emanuel Jauk, Mathias Benedek, Karl Koschutnig, Gayannée Kedia, Aljoscha C. Neubauer

**Affiliations:** University of Graz/Austria, BioTechMed Graz, Graz, Austria

## Abstract

Subclinical narcissism is a personality trait with two faces: According to social-cognitive theories it is associated with grandiosity and feelings of superiority, whereas psychodynamic theories emphasize vulnerable aspects like fluctuating self-esteem and emotional conflicts. The psychodynamic view, however, is commonly not supported by self-report studies on subclinical narcissism. Personality neuroscience might help to better understand the phenomenon of narcissism beyond the limits of self-report research. While social-cognitive theory would predict that self-relevant processing should be accompanied by brain activity in reward-related areas in narcissistic individuals, psychodynamic theory would suggest that it should be accompanied by activation in regions pointing to negative affect or emotional conflict. In this study, extreme groups of high and low narcissistic individuals performed a visual self-recognition paradigm during fMRI. Viewing one’s own face (as compared to faces of friends and strangers) was accompanied by greater activation of the dorsal and ventral anterior cingulate cortex (ACC) in highly narcissistic men. These results suggest that highly narcissistic men experience greater negative affect or emotional conflict during self-relevant processing and point to vulnerable aspects of subclinical narcissism that might not be apparent in self-report research.

## Introduction

The ancient myth of narcissus comes in several different versions. In Ovid’s classic version, the beautiful young hunter Narcissus, who rejects the love of the nymph Echo, is deemed by the gods to fall in love with his mirror image. Fully entranced by his own reflection in a pool of water, Narcissus eventually realizes that his love cannot be reciprocated, which leads him to commit suicide. In another prominent version by Pausanias, the myth has a different ending: Narcissus is gazing at himself, when suddenly a leaf falls into the water and distorts the image. Narcissus is shocked by the ugliness of his mirror image, which ultimately leads him to death.

These two versions of this ancient myth relate to the “two faces” of narcissism, namely grandiose and vulnerable narcissism^1^. Subclinical manifestations of narcissism are commonly conceptualized in terms of grandiose narcissism, which circumscribes feelings of superiority, exaggerated self-worth, and entitlement^2^. In contrast, narcissistic vulnerability is expressed in hypersensitivity, anxiety, and pronounced self-monitoring^3^. Psychodynamic theory posits that narcissistic grandiosity is always accompanied by vulnerable aspects, which appears to be supported by clinical research (e.g. ref. 4). In nonclinical personality research, however, measures of grandiose and vulnerable narcissism display small^5^ or zero correlation^1, 3^. Grandiose narcissism is associated with higher explicit^6^ and implicit self-esteem^7^, which might be the cause of higher self-reported psychological well-being^8^. However, narcissism is also associated with instability of self-esteem in terms of greater dependence upon everyday interpersonal events^9^. This might relate to emotion regulation difficulties and interpersonal conflicts associated with narcissism, particularly in men (cf. refs 10 and 11).

Developmental theories of narcissism also emphasize either its grandiose or vulnerable aspects. Social-cognitive theory posits that, for the development of subclinical narcissism, no “deficits deep down” are needed, but narcissism rather emerges from (unjustified) parental overvaluation^12, 13^. Consequently, social-cognitive theorists emphasize the view that narcissism is neither a facade to mask latent deficits nor a defense to protect the (fragile) self, but reflects a truly inflated sense of self-worth. This view is supported by studies demonstrating an increase of narcissism in younger birth cohorts due to cultural changes (e.g., ref. 14). In contrast, psychodynamic theory posits that narcissism emerges primarily from unreliable, cold, and not sufficiently empathic parents who exhibit indifference or even aggression towards their children^15, 16^. Empirical tests of both theories show that parental overvaluation is the primary driving force of subclinical narcissism, but parental coldness also plays a major role^12, 17^.

According to behavioral research, subclinical narcissism seems to be associated with psychological health rather than developmental deficits, although there is some evidence for emotion regulation difficulties; specifically in men^10, 11^. Grandiose narcissism is largely unrelated to vulnerable aspects in self-report studies, which points to the independence of the “two faces” within subclinical personality variation. Personality neuroscience might help to unveil aspects of narcissism that might not be subject to self-report studies. Recent research shows, for instance, that male grandiose narcissists display increased activity in the “social pain network” (anterior insula, subgenual anterior cingulate, and dorsal anterior cingulate) following social rejection, though they do not report experiencing higher levels of social rejection in self-report measures^18^.

This study set out to explore the neural correlates of narcissistic “self-admiration”, the probably most striking feature of grandiose narcissism. We had extreme groups of high and low narcissistic individuals perform a visual self-recognition paradigm during functional imaging, thus “transferring” the myth of Narcissus into the scanner. This experimental procedure was not only chosen because it nicely resembles the ancient myth, but also because visual-self recognition triggers various aspects of self-referential emotional processing that might not be accessible to self-report. As this is the first study to directly address the neural correlates of visual self-recognition associated with narcissism, it is largely explorative in nature, and no exact regions of interest can be defined a-priori. However, relevant psychological processes and their respective neural substrates can be inferred from social-cognitive vs. psychodynamic theories of narcissism: If narcissism reflects a truly exaggerated sense of self-worth in terms of self-admiration, visual self-recognition should be accompanied by brain activation reflecting reward and/or liking, as it is the case for high self-esteem^19^. Following this “self-reward” - hypothesis, one might expect activation in the dopaminergic system, specifically in subcortical regions^20^. If narcissists display latent deficits in self-esteem and emotion regulation as psychodynamic theory suggests, visual self-recognition should go along with neural activity indicating negative affect or conflicting emotional processing (anterior cingulate^18^). Of course, both aspects could also hold true simultaneously. This study hence undertakes an empirical test of opposing assumptions regarding the psychological mechanisms underlying narcissism as posited by social-cognitive and psychodynamic theories.

## Method

### Participants and Material

The sample consisted of extreme groups of high (*n* = 21) and low (n = 22) narcissistic participants selected out of a large pool (*N* > 600) of individuals who were pre-screened with a German version of the Narcissistic Personality Inventory (NPI; ref. 2). The NPI assesses narcissism as a continuous trait using 40 forced-choice statements such as “I think I am a special person” vs. “I am no better or worse than most people” and is conceived the long-time standard self-report measure of narcissism^21^. It is based on the diagnostic criteria for clinical narcissism (2; for latest version, see ref. 22) and thus focuses on grandiose aspects of narcissism such as authority, exhibitionism, and superiority^23^. NPI scores are, in the general population, uncorrelated with measures exclusively designed to assess vulnerable aspects of narcissism^3^.

Upon arrival at the lab, participants were re-assessed using a Likert-type adaptation of the NPI (see also ref. 24), which allowed for intermixing the items with a Big Five personality scale^25^ in order to prevent participants from guessing the study aim. Additionally, participants completed the German Multidimensional Self-Esteem Scale^26^ and other personality measures that are not analyzed here. Only individuals who scored in the lower or upper tertile of the NPI at both measurements (screening and first lab session; timespan of at least one month) were classified as stable non-/narcissists and were invited to take part in this study. To address the possibility that the low-narcissism group might display below-average self-esteem (i.e., possibly pointing in the direction of anxious personalities, which would not be an adequate control group), we compared sample means with the sex-specific norms^26^. Self-esteem of the low narcissism groups did not deviate from the respective population mean (*t*_13 women_ = −1.29, *p* = 0.220; *t*_7 men_ = −1.02; *p* = 0.341). In the high narcissism groups, women showed higher self-esteem (*t*_7 women_ = 4.83; *p* = 0.002), but men did not (*t*_12 men_ = 1.60; *p* = 0.135). All participants were right-handed, heterosexual, did not report any history of neurological or mental disorders, and gave written informed consent to the study. Sex was counterbalanced across the high and low narcissistic groups. Table 1 displays personality characteristics of the two experimental groups. The experimental protocol was approved by the ethics committee of the University of Graz (GZ. 39/28/63 ex 2013/14) and carried out in accordance with the relevant guidelines and regulations.

**Table 1:** Sample Characteristics.

	Women (*n* = 22)	Low Narcissism (*n* = 14)	*t*_*(df)*_	*d*	*p*	Men (*n* = 21)	Low Narcissism (*n* = 8)	*t*_*(df)*_	*d*	*p*
	High Narcissism (*n* = 8)					High Narcissism (*n* = 13)				
Narcissism	3.13 (0.24)	2.24 (0.22)	8.87 _(20)_	3.97	<0.001	3.13 (0.16)	2.11 (0.24)	11.64 _(19)_	5.34	<0.001
Self-Esteem	5.63 (0.44)	4.63 (0.72)	3.54 _(20)_	1.58	0.002	5.57 (0.85)	4.82 (1.02)	1.81 _(19)_	0.83	0.086
Neuroticism	1.88 (0.48)	2.40 (0.58)	−2.14 _(20)_	−0.96	0.045	2.04 (0.73)	2.28 (0.73)	−0.74 _(19)_	−0.34	0.469
Extraversion	3.75 (0.33)	2.90 (0.64)	4.15 _(19.88)_	1.86	0.001	3.50 (0.43)	2.53 (0.62)	4.23 _(19)_	1.94	<0.001
Openness	3.40 (0.44)	3.40 (0.42)	0.00 _(20)_	0.00	1.00	3.77 (0.26)	3.25 (0.59)	2.35 _(8.64)_	1.60	0.045
Agreeableness	1.91 (0.60)	2.59 (0.45)	−3.06 _(20)_	−1.37	0.006	1.92 (0.60)	2.25 (0.60)	−1.22 _(19)_	−0.56	0.239
Conscientiousness	3.44 (0.53)	2.95 (0.75)	1.62 _(20)_	0.72	0.121	3.33 (0.33)	2.78 (0.62)	2.65 _(19)_	1.22	0.016
Age	24.25 (4.20)	23.43 (3.55)	0.49 _(20)_	0.22	0.630	23.38 (2.60)	24.63 (3.50)	−0.93 _(19)_	−0.43	0.363
PAext_Self_	4.47 (1.19)	4.48 (1.08)	−0.03 _(20)_	−0.01	0.979	4.04 (0.87)	3.91 (0.61)	0.38 _(19)_	0.17	0.711
PAext_Friend_	4.06 (0.88)	4.16 (1.26)	−0.19 _(20)_	−0.08	0.848	3.94 (1.05)	4.03 (0.69)	−0.21 _(19)_	−0.10	0.834
PAext_Stranger_	4.47 (1.09)	4.57 (0.99)	−0.23 _(20)_	−0.10	0.824	4.06 (0.82)	3.88 (0.61)	0.54 _(19)_	0.25	0.595
ΔPAext_Self-Friend_	0.41 (1.37)	0.32 (1.37)	0.14 _(20)_	0.06	0.890	0.10 (0.99)	−0.13 (0.88)	0.52 _(19)_	0.24	0.610
ΔPAext_Self-Stranger_	0.00 (0.30)	−0.09 (0.25)	0.75 _(20)_	0.34	0.463	−0.02 (0.22)	0.03 (0.09)	−0.63 _(19)_	−0.29	0.539
PApart_Self_	4.13 (1.25)	3.93 (1.07)	0.39 _(20)_	0.17	0.701	4.54 (0.88)	3.75 (1.39)	1.44 _(10.49)_	0.89	0.179
PApart_Friend_	5.25 (1.67)	4.79 (1.25)	0.74 _(20)_	0.33	0.467	4.46 (1.39)	4.88 (1.55)	−0.63 _(19)_	−0.29	0.534
PApart_Stranger_	4.13 (2.03)	4.50 (1.02)	−0.49 _(9.06)_	−0.33	0.637	4.46 (0.88)	4.25 (1.58)	0.40 _(19)_	0.18	0.696
ΔPApart_Self-Friend_	−1.13 (1.73)	−0.86 (1.35)	−0.41 _(20)_	−0.18	0.690	0.08 (1.32)	−1.12 (1.13)	2.14 _(19)_	0.98	0.046
ΔPApart_Self-Stranger_	0.00 (1.31)	−0.57 (1.50)	0.87 _(20)_	0.39	0.381	0.08 (1.19)	−0.50 (1.93)	0.85 _(19)_	0.39	0.404
Task Accuracy (%)	96.53 (00.03)	97.95 (00.02)	−1.47 _(20)_	−0.66	0.158	97.72 (0.02)	99.42 (0.01)	−2.70 _(15.77)_	−1.34	0.016
Task Accuracy_Self_ (%)	97.57 (00.05)	97.62 (00.03)	−0.03 _(20)_	−0.01	0.977	98.72 (00.03)	98.96 (00.02)	−0.19 _(19)_	−0.09	0.850
Task Accuracy_Friend_ (%)	97.22 (00.03)	98.61 (00.02)	−1.37 _(20)_	−0.61	0.185	97.44 (00.04)	100.00 (00.00)	−2.52 _(12.00)_	−1.45	0.027
Task Accuracy_Stranger_ (%)	94.79 (00.05)	97.62 (00.03)	−1.70 _(20)_	−0.76	0.105	97.01 (00.02)	99.31 (00.01)	−2.49 _(19)_	−1.14	0.022

*Note*. PAext = Physical Attractiveness, external ratings; PApart = Physical Attractiveness, participant’s ratings. Corrected *df* were used in case of unequal variances.

### Design and Procedure

Our main aim was to investigate brain activation differences between high and low narcissists during visual self-face recognition. As common in visual self-recognition research (e.g., ref. 27), we sought to control for important confounds by employing three experimental conditions in the fMRI paradigm: *Self* (own face), *Friend* (a close same-sex friend’s face), and *Stranger* (a same-sex stranger’s face matched for physical attractiveness).

**Figure 1:**
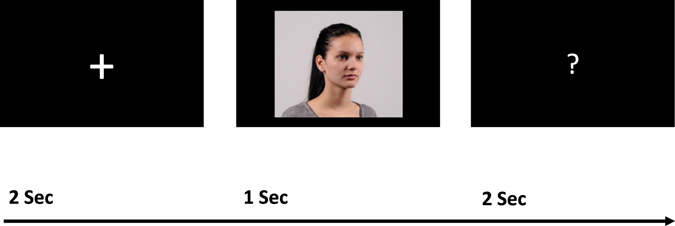
Schematic time course of the experimental paradigm. Participants saw faces of themselves, a friend, or a stranger and were asked to indicate the direction of the face (left or right) when the questionmark sign appeared. The depicted person served as a participant’s friend and did not take part in the experiment; written permission was obtained.

Participants were kept blind to the study’s aim by telling a cover story based on visual self-recognition and personality. The experiment encompassed two sessions: At the first session, participants were invited to the University’s photo studio and were asked to bring a close, same-sex friend (cf. ref. 19). In most cases, this person was the participant’s “best friend”, thus controlling social distance in an ipsative manner (i.e., personal minimum of social distance). Both were instructed to come to the lab in their everyday outfit wearing usual makeup etc. They completed the NPI (intermixed with a German Big Five inventory; see above) and were photographed by a professional photographer. Participants were instructed to display a neutral facial expression and not to wear eyeglasses. We took 72 photographs of each individual from 24 different horizontal and three different vertical angles under standardized lighting conditions. Figure 1 shows a sample photo. Photographs were rated for physical attractiveness by six independent raters (α = 0.88). The independent ratings did not show significant differences between *Self* and *Friend* or *Self* and *Stranger* photographs (PA_*Self*_ = 4.24 [0.97], PA_*Friend*_ = 4.05 [1.01], PA_*Stranger*_ = 4.27 [0.92]; *t*_42 *Self-Friend*_ = 1.06, *d* = 0.19, *p* = 0.297; *t*_42 *Self-Stanger*_ = −0.84, *d* = −0.03, *p* = 0.404). Table 1 provides sex-split external PA ratings and group comparisons within sexes; there were no significant differences between narcissism groups.

At the second lab session, participants underwent the fMRI experiment. After initial instruction outside the scanner, the fMRI session consisted of three blocks encompassing 36 trials each in which participants were presented twelve *Self*, *Friend*, and *Stranger* photographs (with the latter being matched for physical attractiveness) in different horizontal and vertical angles. Conditions were randomized within each block. Blocks were separated by resting periods of 20 sec. The total duration of the MRI experiment was about 10 min.

Figure 1 depicts the time course of a single trial, which consisted of a fixation period (jittered to an average of two seconds within subjects), followed by a one-second presentation of a photograph (*Self*/*Friend*/*Stranger*), and a two-seconds response period, in which participants were asked to indicate via button press whether the face was oriented left or right. This task was chosen to ensure that participants’ attention was directed to the experimental paradigm without induction of evaluative processes on a conscious level. Self-photographs were mirrored at the vertical axis to preserve the natural feeling of gazing into a mirror (e.g., ref. 28).

After completion of the MRI session, participants were asked to rate the physical attractiveness of *Self*, *Friend*, and *Stranger* photographs on a 7-point scale. *Self* and *Stranger* photos were rated as equally attractive by high (*t*_20_ = 0.18, *p* = 0.86, *d* = 0.04) and low narcissists (*t*_21_ = −1.57, *p* = 0.13, *d* = −0.47), and this difference was equal across groups (see Table 1), thus further validating our matching procedure. We also performed sex-split analyses as sex is known to be an important distinguishing factor in narcissism (cf. refs 10 and 11). These revealed that low narcissistic men viewed themselves as less attractive than their friends; strangers were perceived as equally attractive in both sexes (see Table 1). It is important to note that this differences concerns subjective declarations and/or impressions, while there was no objective difference in the external ratings (see above). Finally, participants were debriefed and asked whether they had guessed the study aim. One participant (high narcissist, male) did so and was thus excluded from all analyses.

### fMRI Data Acquisition

Whole brain imaging was performed on a 3 T Siemens Skyra MRI system (Siemens Medical Systems, Erlangen, Germany) using a 32-channel head coil. T2*-weighted functional images were acquired using a single shot gradient echo planar imaging (EPI) sequence (TR = 1850 ms, TE = 30 ms, flip angle = 90°, 32 transversal slices, 3.5 mm isomorphic with distance factor 20%, interleaved slice ordering). Head motion was corrected online. The first two volumes of each scan were discarded in order to allow for T1 equilibration effects. Head motion was restricted using firm padding surrounding participant’s heads. Visual stimuli were presented using the software Presentation (Neurobehavioral Systems, Albany, CA) onto a computer screen and viewed through a mirror attached to the head coil.

### Analysis Plan

For the fMRI analyses, our main contrast of interest was the conjunction of *Self* > *Friend* & *Self* > *Stranger*. This contrast reflects brain activation specific to viewing one’s own face versus someone else’s face while controlling for potential differences in familiarity (*Friend*) and physical attractiveness (*Stranger*). To check for the validity of the experimental protocol, we first performed a whole-brain analysis of this contrast in the full sample, which should unveil regions that are known to be implicated in self-viewing^29, 30^. In the next step, we used them as regions of interest (ROIs), for comparisons of brain activation (i.e., signal change) between high and low narcissism groups. Sex was considered as an additional moderating factor because male and female narcissism are commonly found to differ, particularly when it comes to emotion regulation^10, 11^. Additionally, we examined the effect of self-viewing in group-specific analyses to test the robustness of effects across narcissism groups.

### fMRI Data Analyses

Functional MRI data analysis was performed using SPM 8 software (Wellcome Department of Imaging Neuroscience, London, UK) and the rex toolbox (version 2.1). Preprocessing steps included slice time acquisition correction, realignment, spatial normalization to an averaged EPI template in standard Montreal Neurological Institute (MNI) space, and smoothing with a 10-mm full-width at half-maximum Gaussian kernel.

We specified first level models by regressing voxelwise BOLD on the conditions *Self*, *Friend*, and *Stranger*. Additionally, horizontal and vertical viewing angle and left/right-orientation of the images were included as trial-by-trial regressors. Six motion parameters were specified as additional regressors of no interest. Linear contrasts were used to obtain subject-specific (1^st^ level) estimates for the effects of *Self* > *Friend* and *Self* > *Stranger*. These contrasts were then entered as within-subjects factors in a 2^nd^ level flexible factorial design, where the main effect of interest represents the conjunction *Self > Friend* & *Self > Stranger*. Note that these contrasts are dependent, as *Friend* and *Stranger* conditions were subtracted from *Self* in each contrast. SPM orthogonalizes the respective conjunction analysis in the case of non-orthogonal contrasts (for details, see refs 31 and 32) which was not the case for these contrasts. *Narcissism* (high/low) was included as a between-subjects factor. Correction for multiple comparisons was performed by means of family-wise error (FWE) – correction at voxel level (*p*_FWE_ < 0.05) with a minimum of k = 3 contiguous voxels and false discovery rate (FDR) at cluster level (*p*_FDR_ < 0.05).

### Data availability statement

The study data can be obtained via the first author upon request.

## Results

**Figure 2:**
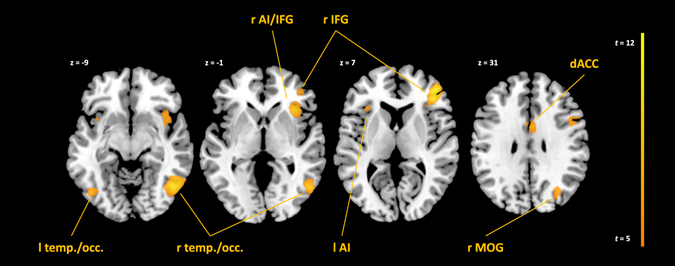
Conjunction *Self*> *Friend* & *Self* > *Stranger*, full Sample, *p*_FWE_ < 0.05. l = left, r = right. AI = anterior insula, IFG = inferior frontal gyrus, dACC = dorsal anterior cingulate cortex, MOG = middle occipital gyrus. Plane identifiers are in MNI space.

We first evaluated the validity of our experimental protocol by a whole-brain analysis of *Self*-specific effects in the full sample. The findings should resemble meta-analytic effects of visual self-recognition^29, 30^. The conjunction analysis of *Self* > *Friend* & *Self* > *Stranger* revealed a pattern of predominantly right-hemispheric clusters. Viewing one’s own face (as compared to faces of friends and strangers) was accompanied by increased brain activation in clusters in the right and left inferior frontal gyri (IFG) and anterior insular cortices, a cluster in the dorsal anterior cingulate cortex (ACC), as well as several mainly right-hemispheric temporo-occipital clusters (extending to the fusiform gyri), and parieto-occipital clusters (see Fig. 2 and Supplementary Table 1). This activation pattern closely resembles meta-analytic findings for visual self-recognition^29, 30^, thereby supporting the validity of our experimental procedure. We also observed a small cluster in the midbrain (ventral tegmental area; see Supplementary Table 1), which is not commonly implicated in self-face processing, but was previously reported in terms of dopaminergic activity during visual self-face *evaluation* ^19^.

In a next step, we tested whether brain activation during viewing one’s one face differs between people of high versus low narcissism. We conducted between-subjects analyses for functionally defined ROIs obtained from the analysis in the full sample within clusters that survived error correction by means of FDR (see Supplementary Table 1). Although the midbrain cluster did not meet this conservative criterion, it was also subjected to ROI analyses because of a-priori interest in the dopaminergic system (see above). Individual signal change estimates were subjected to ANOVAs encompassing the within-subjects factor *condition* (*Self* > *Friend*/*Self* > *Stranger*) and the between-subject factors *narcissism* (high/low) as well as *sex* (female/male). Sex was considered a moderating factor because previous research indicated that female and male narcissists differ at psychological^10, 11^ and also neurophysiological levels^33, 34^. Male narcissists displayed higher brain activation in the dorsal ACC (interaction *narcissism* * *sex*, *p* = 0.043, *η* ^2^_part._ = 0.101), while the main effects of condition, narcissism, and sex were not significant in this cluster (*p*s = 0.258, 0.366 and 0.915, respectively). A similar finding also emerged in the right temporo-occipital cortex by trend (interaction *narcissism* * *sex*, *p* = 0.096*, η* ^2^_part._ = 0.069). We observed no significant main effects or interactions of *narcissism* and *sex* in the other clusters, including the midbrain (see Supplementary Table 3), confirming the specificity of the experimental procedure.

**Figure 3:**
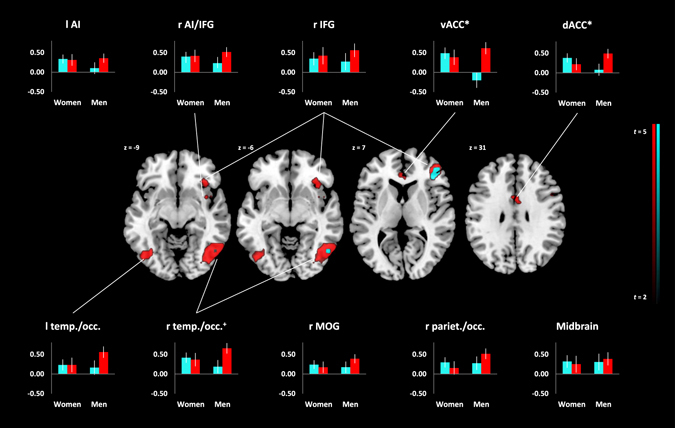
Conjunction *Self* > *Friend* & *Self* > *Stranger*, whole brain analyses within subsamples (red = high narcissism, cyan = low narcissism.), *p*_FWE_ < 0.05. Graphs display interaction narcissism*sex in regions of interest, ^+^ *p* < 0.10, *p < 0.05; vertical axes indicate signal change, error bars denote +/−1 *SE* of the mean. l = left, r = right. AI = anterior insula, IFG = inferior frontal gyrus, v/dACC = ventral/dorsal anterior cingulate cortex, MOG = middle occipital gyrus. Plane identifiers are in MNI space.

We observed similar effects for self-viewing in separate analyses for high and low narcissism groups as for the total sample, though the effects were generally stronger in the high narcissistic group (see Fig. 3 and Supplementary Table 2). In the high narcissistic group, there was an additional peak in the ventral ACC that was not evident in the full sample, why we also conducted a functional ROI analysis in this region. We observed a significant main effect of *narcissism* in the ventral ACC (*p* = 0.045, *η* ^2^_part._ = 0.099), which was significantly moderated by the interaction narcissism * sex (*p* = 0.011, *η* ^2^_part_ = 0.154) in the way that male narcissists exhibited higher brain activation (see Fig. 3 and Supplementary Table 3). In complemental whole brain and ROI analyses, we also checked for effects of self-esteem and reaction time (button press in experimental paradigm), which did not alter the results substantially. Supplementary Table 4 provides ROI analyses statistically corrected for individual differences in self-esteem; the effects in the dACC and vACC remained significant after correction (interaction narcissism*sex, *p*_dACC_ = 0.050 and *p*_vACC_ = 0.009, respectively).

## Discussion

This is the first published study to examine the neural correlates of narcissistic “self-admiration” using fMRI. We conducted a carefully designed experiment in which extreme groups of high and low narcissism (assessed with the NPI, the long-time standard measure of subclinical narcissism), viewed images of themselves, close friends, and similarly attractive strangers. By these means, we sought to uncover the neural responses of visual self-recognition in narcissistic individuals, which might help to better understand the phenomenon of narcissism beyond the limits of self-reports (cf. ref. 18). Based on social-cognitive and psychodynamic theories of narcissism, we expected that highly narcissistic individuals would either display neural activation indicating self-gratification (subcortical dopaminergic regions; refs 19, 20) or negative affect and conflicting emotional processing (anterior cingulate^18^).

Our results support the hypothesis of negative affect rather than self-admiration: Highly narcissistic men displayed increased activation in the dorsal and ventral ACC. While anterior midline regions are known to be generally involved in self-referential processing^29, 35^, interindividual differences in d/vACC activation might be associated with more specific psychological processes: The dorsal ACC is a key region in conflict monitoring^36^, expectancy violation^37^, and negative affect^38^. All of these can be considered relevant to self-referential processing (which implies *evaluative* processing; see below), but their actual degree of involvement might depend upon interindividual differences. For example, the dorsal ACC has been associated with the experience of social exclusion in individuals with low self-esteem^39^. Recently, in a similar vein, Cascio and colleagues^18^ reported that narcissistic individuals display increased activation in the “social pain network” (dorsal ACC, subgenual ACC, and anterior insula) following social exclusion in a cyberball paradigm. Importantly, highly narcissistic individuals did not report elevated feelings of social exclusion in a self-report measure, which lead the authors to conclude that “narcissists’ social pain [is] seen only in the brain” (p. 335). Visual self-recognition may induce similar distress in narcissistic individuals, because narcissists might engage in greater self-monitoring, which is consistent with the adaptive control hypothesis of ACC function^38^. Interestingly, dACC activation is also associated with self-viewing when being observed by others, which may lead to the experience of embarrassment, in which “the ACC might […] serve as a hub, integrating information about the reflective self that is used in evaluating perceptual self-face images” (ref. 40, p. 570).

The view that narcissistic self-processing is accompanied by negative affect or emotional conflict is further substantiated by the activation in the ventral ACC in highly narcissistic individuals. The ventral ACC is specifically involved in processing *negative* self-referential material^41^, particularly when this material is *self-relevant* ^42^. This implies that visual self-recognition is a potentially threatening situation to narcissistic individuals, which are known to be overly sensitive to ego-threat^43^.

Grandiose narcissism thus may encompass vulnerable aspects as well, though these might not be apparent in self-report research. Our results point to subclinical narcissism being not qualitatively but rather quantitatively different from clinical narcissism, for which “many contemporary clinical experts on narcissistic personality disorder now recognize that grandiose self-states oscillate or co-occur with vulnerable self-states and affective dysregulation” (ref. 4, p. 428).

A recent structural diffusion tensor imaging study found that narcissism goes along with weakened frontostriatal connectivity of white matter tracts^44^. The authors interpret their findings in terms of a neural disconnect between brain regions responsible for self-representation (medial frontal cortex) and reward (ventral striatum). This finding might help to understand why one of our initial hypotheses, the “self-reward-hypothesis”, did not hold true: Narcissists seem to habitually lack an *intrinsic* system for self-rewarding activity, why they strive for reward from their external (social) environment^11^. In order to receive reward in terms of positive social feedback, highly narcissistic individuals must be very concerned about their (visual) self-presentation^45^. Narcissists thus have a pronounced disposition to self-evaluation, which may lead to increased (voluntary or involuntary) self-monitoring^46^. This explains why highly narcissistic individuals display neural activation that points to negative affect in the course of evaluative processes rather than self-reward and/or liking (which appears to be intrinsically reduced on a brain structural level). Considering the two versions of the ancient myth of Narcissus, our results are in favor of the less prominent version, in which Narcissus is shocked to death by the ugliness of his mirror image when a leaf drops into the water. This myth can be seen to metaphorically reflect the ongoing critical self-monitoring that narcissists display when confronted with self-relevant material, presumably due to a lowered intrinsic coupling between self-representation and self-reward/liking.

Activation differences pointing to expectancy violation and affective dysregulation were apparent only in men, but not in women, in this study. It has long been hypothesized that narcissism might qualitatively differ between men and women, with men displaying more emotionally dysfunctional characteristics (e.g., ref. 11). Recent empirical evidence indicates that male narcissists display lower performance on measures of emotional intelligence, which is not the case in women^10^. Most strikingly, it was also found that narcissism is accompanied by general elevations in cortisol levels and exaggerated physiological stress responses, but only in men^33, 34^. Taken together with our fMRI findings, these results underpin the presumption that narcissism is qualitatively different in women and men, with primarily men showing increased sensitivity to potentially threatening situations and maladaptive affective regulation. The question remains, however, why our experiment – like previous ones^33, 34^ – did not unveil any dissociation between high and low narcissistic women. Reinhard and colleagues^34^ argued that sex differences in distress indicators associated with narcissism (higher basal cortisol levels in men) might be explained along different narcissistic strategies associated with male and female gender roles: While masculinity is associated with independence and agency, thus promoting individualism over the acceptance of social support, femininity encourages seeking social support. By this means, women might, on average, develop higher resilience towards potentially stressful situations. Future studies could investigate gender roles as potentially mediating variables between sex and physiological outcomes associated with narcissism; it might be the case that women with a more masculine attitude display similar effects as men do. Also, considering gender roles might help to unveil other – probably more subtle – behavioral and neural mechanisms associated specifically with female narcissism.

There are some limitations to this study. Most notably, the sample under study was rather small, but nonetheless carefully selected with respect to large (about 4 *SD*) and stable differences in narcissism. Using these extreme groups and a two-step fMRI data analysis procedure (whole brain and ROI analyses), it was possible to obtain robust results that satisfy the most conservative statistical criteria. Another limitation can be seen in that this study focused on grandiose narcissism, though increasing efforts are devoted to the study of vulnerable narcissism as an independent trait in narcissism research. However, our results strengthen the notion that grandiose narcissism entails vulnerable aspects when it comes to involuntary neurophysiological reactions, which points to a general mechanism underlying both traits. Future studies could use the experimental paradigm in an extended design encompassing grandiose and vulnerable aspects of narcissism to further elucidate their similarities and differences.

Finally, it shall be acknowledged that ACC activation cannot unambiguously be attributed to any single mental process^36^. As outlined above, the (d) ACC is generally involved in (visual or non-visual) self-processing^29^, but has also been associated with conflict monitoring, expectancy violation, pain, and negative affect^36–38^. Since processing of self-referential information might always involve some degree of self-evaluation, and thus, conflicting emotional processing or negative affect, these functions might not be as different as they might seem in the first place. In line with this, it has been proposed that the dACC might act as a hub integrating information for self-evaluative processing^40^. Importantly, though the ACC is generally involved in self-processing, the *degree* of ACC activation might still relate to interindividual differences^18^. For the interpretation of these interindividual differences, it seems most important that ACC activation is related to processes with *negative* emotional valence, especially in the context of conflict monitoring^38^. However, the ACC has not only been associated with self-referential processing and negative affect, but also with reward (particularly the ventral ACC; ref. 36), which could be seen as supporting the self-reward hypothesis rather than the negative affect/emotional conflict hypothesis. However, we did not observe significant group differences in the more unambiguously reward-related midbrain cluster. In complemental analyses, we also investigated other reward-related areas such as the nucleus accumbens and the caudate nucleus, but did not find any significant activation (small volume correction). Finally, the results observed here closely resemble those of Cascio and colleagues^18^, who related ACC activation in narcissistic individuals to negative affect (social exclusion). Taken together, the data are clearly more in line with the notion of negative affect or emotional conflict than the self-reward hypothesis.

This study set out to explore the neural correlates of narcissistic “self-admiration” within the normal personality variation of narcissism. Contrary to what would be expected on the basis of self-reports, we found that highly narcissistic men display brain activation patterns that point to prevailing negative affect or emotional conflict during visual self-recognition. These results are more in line with psychodynamic than social-cognitive theories on narcissism. While previous social-cognitive research used to focus on voluntary and conscious aspects of narcissism by means of self-report, our neurophysiological results point to latent affective dysregulation in the processing of self-relevant material.

## Electronic supplementary material

Supplementary Tables 1–4
