# Supplementary material for: Self-viewing is associated with negative affect rather than reward in highly narcissistic men: an fMRI study
Source: Sci Rep. 2017 Jul 19;7:5804. doi: 10.1038/s41598-017-03935-y (PMC5517462; doi:10.1038/s41598-017-03935-y)
Supplement: Supplementary file 1 — Supplementary Tables 1–4 [file 41598_2017_3935_MOESM1_ESM.docx]

Supplementary Material to:

Self-viewing is associated with negative affect rather than reward in highly narcissistic men: an fMRI study.

Emanuel Jauk^1^*, Mathias Benedek^1^, Karl Koschutnig^1^, Gayannée Kedia^1^, & Aljoscha C. Neubauer^1^

^1^ University of Graz / Austria, BioTechMed Graz

* *Corresponding Author:*

Emanuel Jauk

Department of Psychology / University of Graz

Universitätsplatz 2

8010 Graz

Austria

emanuel.jauk@uni-graz.at

+43 316 380 5120

Supplementary Table 1

*Whole-brain analysis of Conjunction Self>Friend & Self>Stranger, full sample*

| Region | Peak (MNI) | | |  |  | Cluster |  |
| --- | --- | --- | --- | --- | --- | --- | --- |
|  | x | y | z | *t* | *P*_FWE_ | k | *p*_FDR_ |
| r AI/IFG | 45 | 42 | 10 | 11.94 | <.001 | 244 | <.001 |
| r temp./occ. | 52 | -67 | -5 | 10.40 | <.001 | 189 | <.001 |
| dACC | 3 | 0 | 31 | 7.51 | <.001 | 34 | <.001 |
| l temp./occ. | -43 | -70 | -8 | 7.50 | <.001 | 37 | <.001 |
| r IFG | 52 | 11 | 27 | 7.28 | <.001 | 50 | <.001 |
| r pariet./occ. | 31 | -67 | 31 | 7.14 | <.001 | 84 | <.001 |
| l AI | -33 | 25 | 6 | 6.80 | .001 | 34 | <.001 |
| Midbrain | 3 | -21 | -15 | 6.72 | .001 | 3 | .216 |
| r MOG | 38 | -84 | 13 | 6.54 | .001 | 33 | <.001 |
| l MOG | -36 | -91 | 10 | 6.14 | .005 | 5 | .119 |
| r SMG | 62 | -21 | 38 | 5.59 | .019 | 7 | .073 |

*Note.* Table corresponds to Figure 2 in the manuscript. l = left, r = right. AI = anterior insula, IFG = inferior frontal gyrus, d/vACC = dorsal/ventral anterior cingulate cortex, MOG = middle occipital gyrus, SMG = supramarginal gyrus.

Supplementary Table 2

*Whole-brain analysis of Conjunction Self>Friend & Self>Stranger, within subsamples of high/low narcissism*

| Region | Peak (MNI) | | |  |  | Cluster |  |
| --- | --- | --- | --- | --- | --- | --- | --- |
|  | x | y | z | *t* | *P*_FWE_ | k | *p*_FDR_ |
| High Narcissism |  |  |  |  |  |  |  |
| r IFG | 41 | 42 | 6 | 9.31 | <.001 | 97 | <.001 |
| r temp./occ. | 45 | -67 | -8 | 9.07 | <.001 | 173 | <.001 |
| l temp./occ. | -43 | -70 | -8 | 6.98 | <.001 | 42 | <.001 |
| r AI | 31 | 28 | -1 | 6.97 | <.001 | 38 | .001 |
| dACC | 3 | 4 | 31 | 6.29 | .003 | 14 | .037 |
| vACC | -1 | 35 | 6 | 6.21 | .003 | 6 | .142 |
| r AI | 41 | 7 | -12 | 6.19 | .004 | 10 | .059 |
| r SMG | 62 | -21 | 38 | 6.01 | .005 | 9 | .053 |
| r precentral G. | 59 | 11 | 38 | 6.09 | .005 | 13 | .038 |
| l Insula | -36 | -7 | -5 | 5.77 | .013 | 6 | .142 |
| l OFG | -29 | 35 | -15 | 5.65 | .018 | 5 | .175 |
| Low Narcissism |  |  |  |  |  |  |  |
| r IFG | 45 | 39 | 10 | 7.33 | <.001 | 36 | <.001 |
| r temp./occ. | 52 | -67 | -5 | 6.80 | .001 | 15 | .007 |

*Note.* Table corresponds to Figure 3 in the manuscript. l = left, r = right. IFG = inferior frontal gyrus, AI = anterior insula, d/vACC = dorsal/ventral anterior cingulate cortex, SMG = supramarginal gyrus, OFG = orbitofrontal gyrus.

Supplementary Table 3

*ANOVAs of signal change in Regions of Interest (ROIs)*

| ROI (MNI Peak Coord.) | Cond. | | Cond.*Narc. | | Cond.*Sex | | Cond.*Narc.*Sex | | Narcissism | | Sex | | Narcissism*Sex | |
| --- | --- | --- | --- | --- | --- | --- | --- | --- | --- | --- | --- | --- | --- | --- |
|  | *F* | *p* | *F* | *p* | *F* | *p* | *F* | *p* | *F* | *p* | *F* | *p* | *F* | *p* |
| r AI/IFG (45 / 42 / 10) | 0.000 | .997 | 2.684 | .109 | 0.668 | .419 | 0.000 | .984 | 1.166 | .287 | 0.059 | .810 | 0.897 | .350 |
| r temp./occ. (52 / -67 / -5) | 0.255 | .617 | 0.921 | .343 | 1.480 | .231 | 0.930 | .341 | 1.884 | .178 | 0.037 | .848 | 2.906 | .096 |
| dACC (3 / 0 / 31) | 1.318 | .258 | 0.017 | .898 | 0.324 | .573 | 0.305 | .584 | 0.838 | .366 | 0.012 | .915 | 4.378 | .043 |
| l temp./occ. (-43 / -70 / -8) | 0.092 | .764 | 0.216 | .645 | 1.128 | .295 | 0.462 | .501 | 1.438 | .238 | 0.617 | .437 | 1.456 | .235 |
| r IFG (52 / 11 / 27) | .639 | .429 | 3.224 | .080 | 1.083 | .304 | 0.329 | .569 | 0.888 | .352 | 0.026 | .873 | 0.294 | .591 |
| r pariet./occ. (31 / -67 / 31) | 0.159 | .692 | 1.306 | .251 | 0.401 | .530 | 0.010 | .920 | 0.099 | .754 | 1.230 | .274 | 1.546 | .221 |
| l AI (-33 / 25 / 6) | 0.047 | .830 | 1.006 | .322 | 2.426 | .127 | 0.612 | .439 | 0.075 | .390 | 0.512 | .478 | 1.121 | .296 |
| Midbrain (3 / -21 / -15) | 0.431 | .515 | 0.062 | .805 | 3.325 | .076 | 1.134 | .293 | 0.001 | .972 | 0.103 | .750 | 0.158 | .694 |
| r MOG (38 / -84 / 13) | 0.001 | .970 | 2.329 | .135 | 3.139 | .084 | 0.355 | .555 | 0.323 | .573 | 0.334 | .567 | 1.203 | .279 |
| vACC (-1 / 35 / 6) | 4.028 | .052 | 0.026 | .872 | 0.118 | .734 | 1.655 | .206 | 4.278 | .045 | 1.759 | .192 | 7.086 | .011 |

*Note.* *df* = 1,37 for all analyses. cond. = within-subjects factor condition (Self>Friend / Self>Stranger). narc. = between-subjects factor narcissism (high / low). = left, r = right. AI = anterior insula, IFG = inferior frontal gyrus, d/vACC = dorsal/ventral anterior cingulate cortex, MOG = middle occipital gyrus.

Supplementary Table 4

*ANCOVAs of signal change in Regions of Interest (ROIs), corrected for self-esteem*

| ROI (MNI Peak Coord.) | cond. | | cond.*SE | | cond.*narc. | | cond.*sex | | cond*narc.*sex | | SE | | narcissism | | sex | | narcissism*sex | |
| --- | --- | --- | --- | --- | --- | --- | --- | --- | --- | --- | --- | --- | --- | --- | --- | --- | --- | --- |
|  | *F* | *p* | *F* | *p* | *F* | *p* | *F* | *p* | *F* | *p* | *F* | *p* | *F* | *p* | *F* | *p* | *F* | *p* |
| r AI/IFG (45 / 42 / 10) | 1.250 | .271 | 1.279 | .265 | 0.770 | .386 | 0.754 | .391 | 0.013 | .911 | 1.109 | .299 | 0.181 | .673 | 0.083 | .775 | 1.062 | .309 |
| r temp./occ. (52 / -67 / -5) | 2.838 | .131 | 2.683 | .110 | 0.002 | .961 | 1.722 | .197 | 0.720 | .410 | 1.390 | .246 | 0.388 | .537 | 0.021 | .887 | 3.252 | .079 |
| dACC (3 / 0 / 31) | 0.745 | .393 | 1.092 | .303 | 0.160 | .691 | 0.377 | .543 | 0.217 | .644 | 0.211 | .648 | 1.030 | .317 | 0.007 | .932 | 4.108 | .050 |
| l temp./occ. (-43 / -70 / -8) | 0.384 | .539 | 0.451 | .506 | 0.005 | .943 | 1.172 | .286 | 0.382 | .540 | 0.497 | .485 | 0.479 | .493 | 0.561 | .458 | 1.568 | .218 |
| r IFG (52 / 11 / 27) | 0.849 | .363 | 1.106 | .300 | 1.103 | .300 | 1.180 | .284 | 0.433 | .514 | 0.104 | .748 | 0.426 | .518 | 0.021 | .885 | 0.314 | .579 |
| r pariet./occ. (31 / -67 / 31) | 1.496 | .229 | 1.384 | .247 | 0.197 | .660 | 0.472 | .496 | 0.000 | .996 | 0.567 | .456 | 0.009 | .924 | 1.144 | .292 | 1.674 | .204 |
| l AI (-33 / 25 / 6) | 0.028 | .868 | 0.019 | .892 | 0.865 | .358 | 2.342 | .134 | 0.575 | .453 | 0.071 | .791 | 0.380 | .541 | 0.515 | .477 | 1.133 | .294 |
| Midbrain (3 / -21 / -15) | 0.479 | .493 | 0.362 | .551 | 0.007 | .936 | 3.171 | .083 | 1.214 | .278 | 0.311 | .580 | 0.059 | .809 | 0.086 | .771 | 0.191 | .664 |
| r MOG (38 / -84 / 13) | 1.152 | .290 | 1.166 | .287 | 0.644 | .427 | 3.314 | .077 | 0.466 | .499 | 2.991 | .092 | 0.117 | .735 | 0.268 | .608 | 1.592 | .215 |
| vACC (-1 / 35 / 6) | 0.183 | .671 | 0.535 | .469 | 0.249 | .620 | 0.138 | .712 | 1.476 | .232 | 1.428 | .239 | 1.501 | .228 | 1.915 | .174 | 7.644 | .009 |

*Note.* *df* = 1,37 for all analyses. cond. = within-subjects factor condition (Self>Friend / Self>Stranger). narc. = between-subjects factor narcissism (high / low). = left, r = right. AI = anterior insula, IFG = inferior frontal gyrus, d/vACC = dorsal/ventral anterior cingulate cortex, MOG = middle occipital gyrus.
